# Differential analysis of nutrient intake, insulin resistance and lipid profiles between healthy and premature thelarche Chinese girls

**DOI:** 10.1186/s13052-019-0758-z

**Published:** 2019-12-19

**Authors:** Yueqin Xu, Yan Li, Shuang Liang, Guimei Li

**Affiliations:** 1 Department of Children Healthcare, The First Affiliated Hospital of Shandong First Medical University, No.16766 Jingshi Road, Jinan, 250014 Shandong China; 2 Department of Pediatrics, the Second Hospital of Shandong University, |No. 247 Beiyuan Street, Jinan, 250033 Shandong China; 3 Department of Pediatrics, Shandong Provincial Hospital Affiliated to Shandong First Medical University, No. 9677 Jingshi Road, Jinan, 250014 China

**Keywords:** Premature thelarche, Nutrient intake, Insulin resistance, Lipid profile, Breast development

## Abstract

**Background:**

Premature thelarche (PT) is a benevolent ailment affecting young girls. Multiple factors are reported to correlate with this condition, but the mechanisms responsible for the onset of PT have not yet been fully investigated. This study aimed to evaluate the relationship of nutrient intake, insulin resistance and lipid profile with PT.

**Methods:**

Two hundred sixty-three girls with PT, and 222 healthy girls of similar age were enrolled into this study. Their demographics, Tanner stage of breast development, nutrient intake, insulin resistance and lipid profiles were compared.

**Results:**

Daily protein and fat intakes, insulin resistance parameters including serum insulin-like growth factor 1, fasting glucose to insulin ratio, quantitative insulin check index and homeostasis model of assessment of insulin resistance, as well as serum levels of triacylglycerol, total cholesterol and low-density lipoprotein, were all significantly altered in PT patients. Daily intake of energy and carbohydrate, and serum level of high-density lipoprotein protein were statistically indistinguishable between PT patients and healthy controls.

**Conclusion:**

Chinese girls with PT are potentially insulin resistant, which warrants more clinical attention and further investigation to address the underlying etiology.

## Background

Premature thelarche (PT) usually manifests in girls below the age of 8, as isolated breast development without accelerated growth, early/advanced pubic and axillary hair development, menarche and/or bone maturation. PT is normally a self-limited ailment, but nearly 15% of incidences show rapid escalation to precocious puberty (PP) [1]. To date, the etiology of PT is still controversial, with suggested mechanisms such as transient secretion of estrogens from ovarian follicular cysts [2], transient and partial activation of the hypothalamic-pituitary-gonadal axis caused by excessive secretion of follicle stimulating hormone (FSH) [3, 4], increased breast sensitivity to estrogens [5] or production of estrogens from adrenal precursors [6], as well as increased dietary estrogen from exogenous food contamination [7, 8].

In general, PT is described as an endogenous origin-related disease, especially in infant girls where its onset is attributed to maternal estrogen exposure during pregnancy [9]. Several other factors, such as nutrition, ethnicity, adiposity, psychosocial and socioeconomic conditions, as well as genetic predisposition, may also contribute to PT onset. However, the mechanisms responsible for the onset of PT in later stages of infancy are not thoroughly investigated.

The current study aimed to investigate the correlation of several nutritional and metabolic factors with onset of PT, such as nutrient intake, insulin resistance parameters and serum lipid profiles. The results could provide a potential group of collective factors to more accurately predict the onset of PT.

## Materials and methods

### Ethical statements

The study protocol was designed following guidelines in the Declaration of Helsinki, and approved by the Ethical Committee of Shandong Provincial Hospital Affiliated to Shandong First Medical University. All participants and their parents or guardians have signed written informed consent forms.

### Participants

This study was conducted from November 2015 to September 2017. Girls admitted in Shandong Provincial Hospital Affiliated to Shandong First Medical University, due to complaint of enlargement of breasts before puberty, were enrolled, who were later confirmed with PT diagnosis according to the following inclusion criteria: 1) isolated breast development before the age of 8; 2) between 4 to 8 years of age; 3) bone age/chronologic age ratio < 1.2 [10]; 4) peak luteinizing hormone (LH) < 5 U/L in an exogenous gonadotropin-releasing hormone (GnRH) test [11]. Exclusion criteria were: 1) had a family history of PP or diagnosed of PP (peak LH ≥ 5 U/L and peak LH/ peak FSH ≥ 0.6 in GnRH test); 2) using creams or pills containing estrogen; 3) combined hypothalamus-pituitary abnormality and congenital metabolic disorder. A total of 263 girls with PT were recruited. In parallel, a total of 222 healthy girls of similar age, without history of PT, any other endocrine disorders or no secondary sexual characteristics, were also recruited as the healthy control group. Meanwhile, 28 ICPP and 25 non-ICPP PT patients, according to Consensus Statement for the diagnosis and treatment of central precocious puberty (2015) [12], were also recruited for comparison of nutritional status. All assessments were conducted by investigators blind to the study group assignment.

### Assessment of demographics

The Greulich and Pyle method was employed for bone age assessment [13]. Body mass index (BMI) was calculated using the formula: body mass / (body height)^2^. The pubertal stage was assessed by trained pediatric endocrinologists according to Tanner’s criteria for breast development in girls [14].

### Assessment of serum hormones

Fasting blood samples (10 mL) were taken in an early morning at 8 am after an overnight fast from all participants. Blood samples were immediately collected in test tubes containing 0.1% EDTA, followed by centrifugation and storage at − 80 °C for other analyses. Levels of estradiol, LH and insulin-like growth factor 1 (IGF1) were measured by a Cobas 8000 modular analyzer, using estradiol chemiluminescence immunoassay kit (ADVIA Centaur, Siemens) and third-generation immunochemiluminometric LH assay kit (Roche, IN, USA), respectively, according to manufacturer’s instructions.

### Assessment of nutrient intake

Families of all participants were issued a recall form to keep records of daily food intake of participants within 1 month of study initiation. The recall form used common food categories, such as salt, sugar, oil, pork, fruit and vegetable, and regular household units, such as tea spoons and cups, to measure food intake. Nutrient intake breakdown was analyzed using Nutritionist software 4 (First Databank, USA) modified for Chinese food.

### Assessment of insulin resistance parameters

Fasting blood glucose level was measured using the hexokinase assay, on an AU5800 Chemistry Analyzer (Olympus, Japan). Fasting blood insulin level was measured using a Cobas 8000 modular analyzer and assay kit (Roche, Germany). The quantitative insulin check index (QUICKI) and homeostasis model of assessment of insulin resistance (HOMA-IR) were employed to assess insulin resistance as previous described [15].

### Assessment of lipid profile

Serum levels of total cholesterol (TC), triacylglycerol (TAG), low-density lipoprotein (LDL) and high-density lipoprotein (HDL) were measured using an AU5800 Chemistry Analyzer (Olympus, Japan).

### Statistical analysis

Data were analyzed using GraphPad Prism 7. Distribution of acquired data was assessed to determine normality using Kolmogorov-Smirnov test. Normally distributed data were analyzed by two-tailed student’s t test, and non-normally distributed data were analyzed by Mann-Whitney test. *P* < 0.05 indicates statistical significance.

## Results

This study included 263 girls diagnosed of PT, with 222 healthy girls also recruited as the control group. Between the anthropometric characteristics of the two groups, there were no significant differences in chronology age, bone age or BMI, indicating a comparable baseline for the rest of the study (Table 1).

**Table 1:** Baseline demographics of patients and control subjects

Characteristics	Patients (*n* = 263)	Healthy (*n* = 222)
Chronology age (yr)	5.3 ± 0.7	5.5 ± 0.8 #
Bone age (yr)	6.1 ± 1.1	5.9 ± 1.2 #
BMI (kg/m^2^)	15.1 ± 2.6	15.8 ± 2.1 #

Values were mean ± SD. # *P* > 0.05

**Fig. 1:**
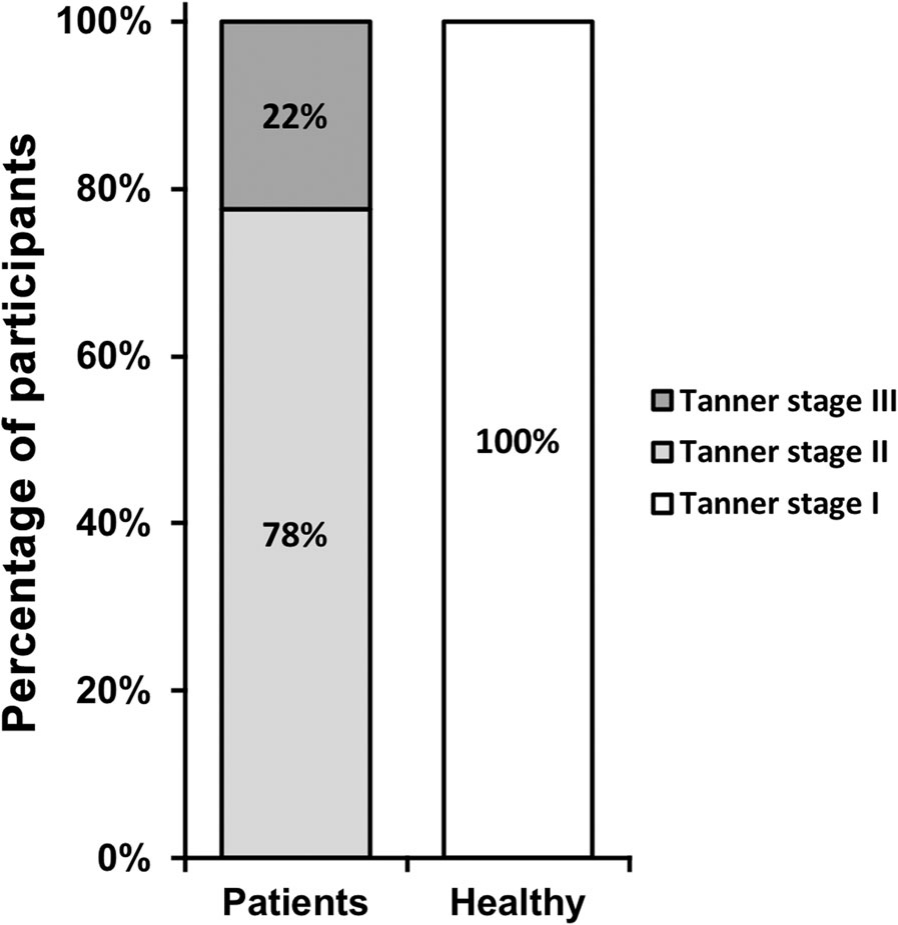
Tanner stage of breast development of PT patients and healthy control

Next, their hormone levels were assessed. Estradiol level in PT patient group was 1.74 ± 0.87 ng/dL, while it was 1.65 ± 0.63 in healthy controls, without statistical difference (*p* > 0.05) (Table 2). However, peak LH was 3.38 ± 0.79 U/L in PT patients, significantly higher than 3.04 ± 0.62 U/L in healthy controls (*p* < 0.05), and peak FSH was 4.07 ± 0.43 U/L in PT patients, also significantly higher than 3.68 ± 0.52 U/L in healthy controls (*p* < 0.05). Similarly, IGF1 was 23.20 ± 0.23 μg/dL in PT patients, significantly higher than 15.50 ± 0.38 μg/dL in healthy controls (*p* < 0.0001) (Table 2). Using Tanner’s criteria to assess breast development in girls in PT patient group, 78% were stage II and the rest were stage III (Fig. 1). On the other hand, all healthy girls were in stage I.

**Table 2:** Baseline hormone levels of patients and control subjects

Characteristics	Patients (*n* = 263)	Healthy (*n* = 222)
Estradiol (ng/dL)	1.74 ± 0.87	1.65 ± 0.63 #
Peak LH (U/L)	3.38 ± 0.79	3.04 ± 0.62 *
IGF1 (ug/dL)	23.20 ± 0.23	15.50 ± 0.38 **
Peak FSH (U/L)	4.07 ± 0.43	3.68 ± 0.52 *

Values were mean ± SD. LH, luteinizing hormone. IGF1, insulin-like growth factor1. *FSH* Follicle stimulating hormone. * *P* < 0.05. *** *P* < 0.0001. # *P* > 0.05

**Fig. 2:**
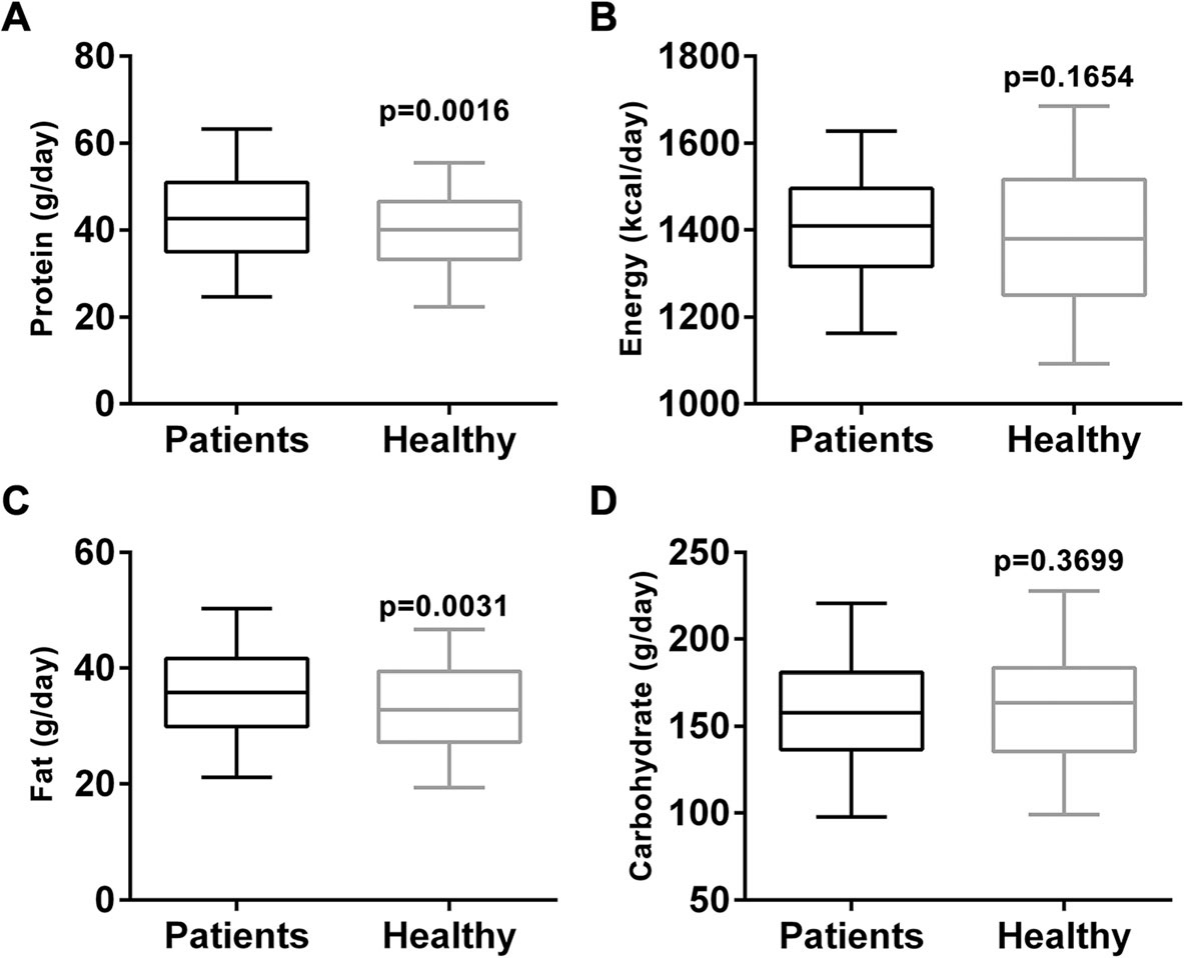
Nutrient intake, including (**a**) Protein, (**b**) energy, (**c**) fat and (**d**) carbohydrate, of PT patients and healthy control. Values were shown as median and 5–95 percentile

Multiple factors were suggested to contribute to the onset of PT, and we started with nutrient intake. Food intake of all participants were recorded for a month, and daily nutrient breakdown was summarized in Fig. 2, in terms of protein (Fig. 2a), energy (Fig. 2b), fat (Fig. 2c) and carbohydrate (Fig. 2d). Daily protein and fat intakes were found to be significantly higher in PT patient group than the healthy group (*p* = 0.0016 and 0.0031, respectively) (Fig. 1a and c). Daily intake of energy and carbohydrate were essentially the same between the two groups (*p* = 0.1654 and 0.3699, respectively).

**Fig. 3:**
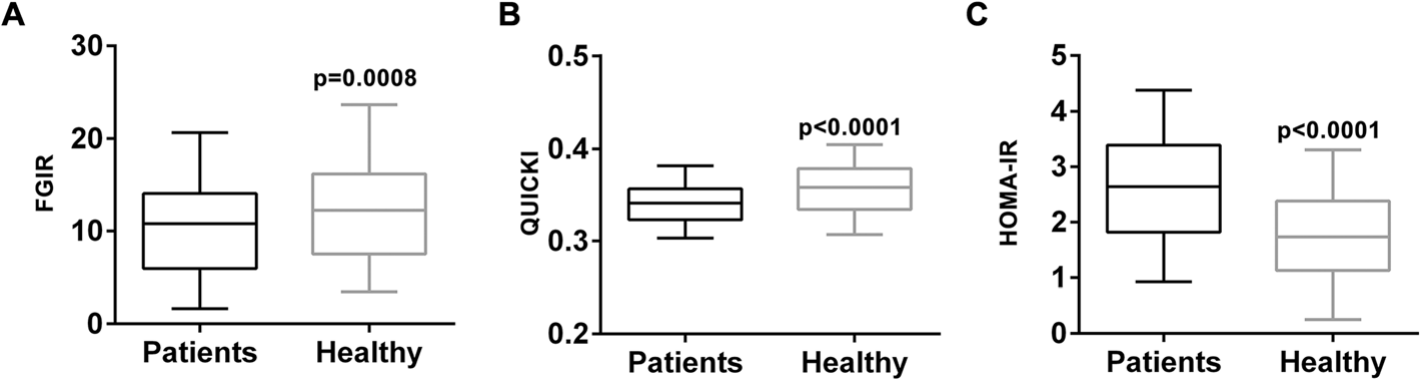
Insulin resistance parameters, including (**a**) fasting glucose to insulin ratio (FGIR), (**b**) quantitative insulin-sensitivity check index (QUICKI) and (**c**) homeostatic model assessment of insulin resistance (HOMA-IR), of PT patients and healthy control. Values were shown as median and 5–95 percentile

The second endpoint assessed was insulin resistance parameters (Fig. 3). Fasting glucose to insulin ratio (FGIR) was slightly lower in PT patient group than the healthy girls, although still statistically significant (*p* = 0.0008) (Fig. 3a). Two other insulin resistance criteria, namely QUICKI and HOMA-IR, were also significantly different between the PT group and the healthy controls (both *p* < 0.0001) (Fig. 3b and c).

**Fig. 4:**
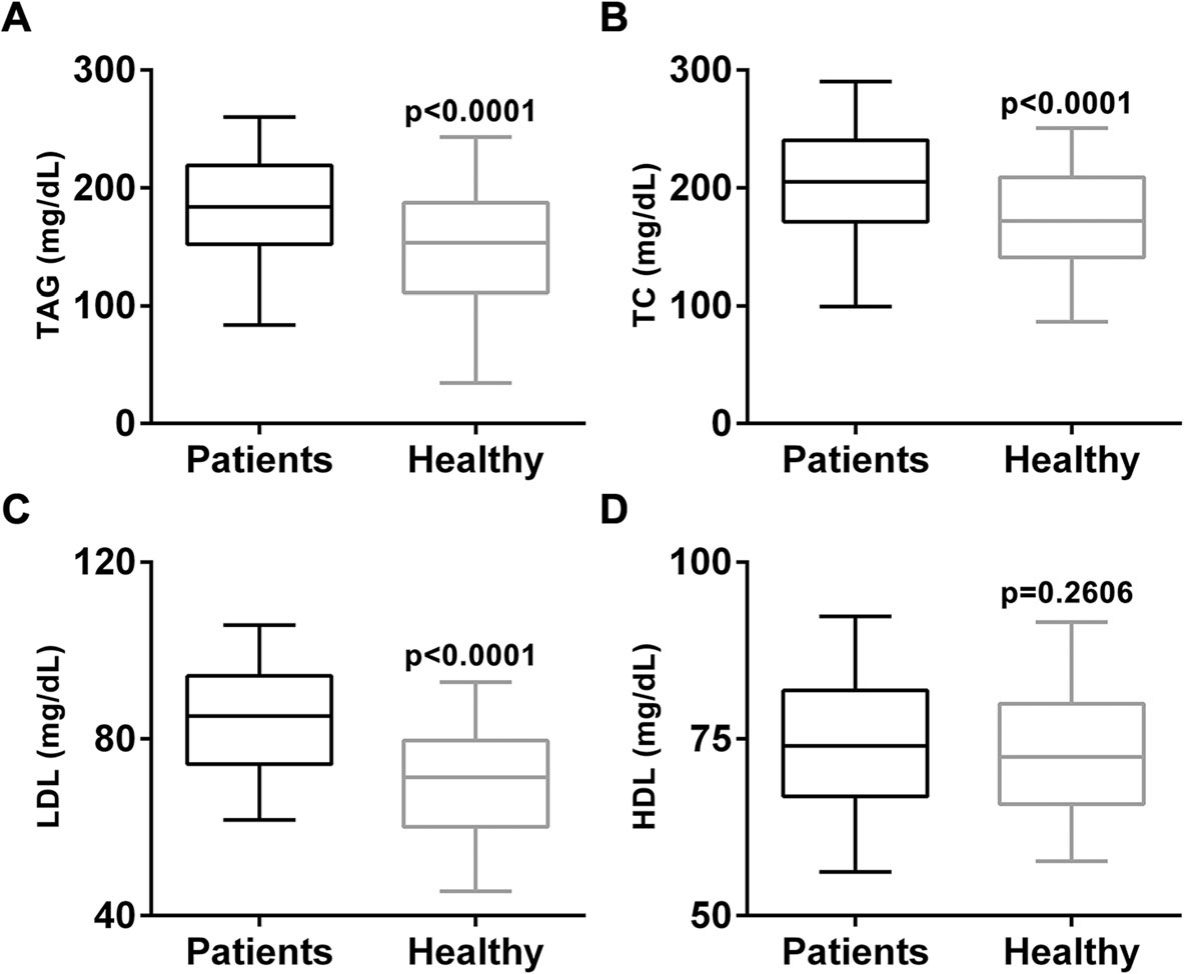
Lipid profile, including (**a**) triacylglycerol (TAG), (**b**) total cholesterol (TC), (**c**) low-density lipoprotein (LDL) and (**d**) high-density lipoprotein protein (HDL), of PT patients and healthy control. Values were shown as median and 5–95 percentile

Lastly, we examined the serum lipid profile of all participants. TC, TAG and LDL were all significantly higher in PT patients than healthy controls (all *p* < 0.0001) (Fig. 4a, b and c). However, HDL of PT patients was slightly but insignificantly higher than healthy girls (*p* = 0.2606) (Fig. 4d).

**Fig. 5:**
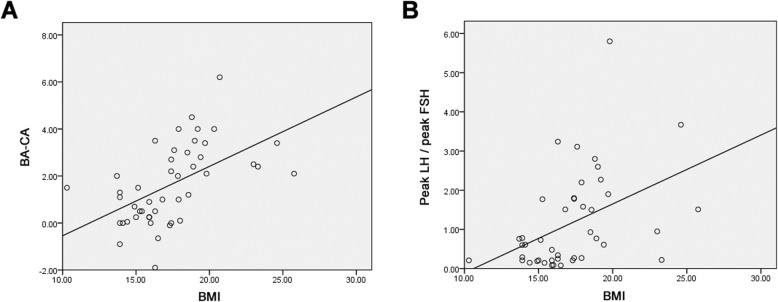
Pearson correlation analysis between BMI and (**a**) BA-CA, (**b**) peak LH / peak FSH among PT patients

At last, Pearson correlation analysis was performed between BMI and BA-CA (bone age minus chronological age) and ratio of peak LH / peak FSH, respectively. In PT patients, BA-CA exhibited a significant positive correlation with BMI (Fig. 5a, *r* = 0.536, *p* = 0.001), and peak LH / peak FSH was also significantly and positively correlated with BMI (Fig. 5b, r = 0.435, *p* = 0.004).

## Discussion

PT in young girls usually manifests as isolated breast hypertrophy in the absence of other sexual maturation, with breast development occurring as early as the age of two [16]. Breast hypertrophy is usually bilateral and in a less proportion of patients unilateral, while the enlargement is not excessive without obvious changes in developments of areolae and/or nipples. Typically, breast hypertrophy does not proceed beyond Tanner stage III, and in unilateral cases it does not proceed beyond Tanner stage II [17], which is in consistence with observations of the current study.

The major aim of the study was to investigate potential risk factors associated with PT, the first of which was nutrient intake. It was reported that the amount of milk, eggs, chicken or fish consumed was not associated with PT [18]. Compared with previous study, instead of analyzing food intake in categories of actual items, nutrient intake was broken down in categories of protein, energy, fat and carbohydrate in the current study. Surprisingly, we found protein and fat intakes were associated with PT, whereas intakes of energy and carbohydrate were not. These findings point out that daily nutrient intake could, to some extent, contribute to onset of PT. However, food intake in the current study was only monitored for a month, and long-term study is needed for more concrete results, especially to establish causal relationship between higher protein and fat consumption and PT incidences.

According to an earlier study, childhood obesity, intra uterine growth retardation, parental obesity and diabetes were associated with PT [19], we therefore also assessed insulin resistance parameters in the participants. Indeed, we observed significantly altered FGIR, QUICKI and HOMA-IR in PT patients, compared to healthy controls, suggesting PT patients are potentially insulin resistant. This is in agreement with a previous case study, where a PT patient was also diagnosed of type II diabetes mellitus, and was therefore resistant to hormone treatment [20]. However, the correlative data of our current study are not sufficient to tell whether insulin resistance is the cause or consequence of PT, and we are looking into further study employing animal models to address this question.

As lipid composition of diabetic patients is usually disrupted [21], we next examined the serum lipid profile of both PT patients and healthy controls. Compared with healthy girls, serum levels of TAG, TC and LDL were significantly elevated, whereas no obvious change in HDL serum level was observed. To date, there has been no study available on lipid composition of PT patients, and our current study provides the first instance to suggest that serum lipid profile is largely altered, at least in Chinese girls with PT. This altered lipid profile is likely a secondary effect of insulin resistance described above, although more investigations are needed to verify this hypothesis.

In addition, using BMI as the indicator of nutritional status, we found BMI displayed significant positive correlations with BA-CA and ratio of peak LH / peak FSH, suggesting higher risk of PT is correlated with better nutritional status (higher BMI). We speculate that over-nutrition could impact the prognosis of PT patients, eventually leading to deficiency in body height and a series of physiological and behavioral problems.

To summarize, data from our study suggest that Chinese girls with PT exhibit elevated potential to develop insulin resistance, which warrants more clinical attention and further investigations to address the underlying etiology. Nevertheless, scope of this study is limited due to its clinical nature, and appropriate animal studies are warranted to uncover the molecular mechanism underlying the observed correlations.

## Conclusions

1. PT is correlated with high intakes of protein and fat.

2. Serum insulin resistance parameters and lipid profile of PT patients are altered.

3. PT and BMI are positively correlated to certain extent.

## Data Availability

All data generated or analysed during this study are included in this published article.

## Abbreviations

PT: Premature thelarche
